# In vivo measurement of the biomechanical properties of plantar soft tissues under simulated gait conditions

**DOI:** 10.1186/1757-1146-5-S1-O39

**Published:** 2012-04-10

**Authors:** Daniel Parker, Glen Cooper, Stephen Pearson, David Howard, Gillian Crofts, Christopher Nester

**Affiliations:** 1 School of Health Sciences, University of Salford, Salford, Greater Manchester, M6 6PU, UK; 2 Manchester Metropolitan University, Manchester, Greater Manchester, M15 6BH, UK

## Background

In vivo biomechanical properties of plantar soft tissues have been assessed via manual indentation using simplified loading profiles [1], or in gait, with low image resolution/capture rates [2]. Since plantar soft tissue properties are highly rate dependent [3] these methods are potentially inadequate. The Soft Tissue Response Imaging Device (STRIDE) permits functionally relevant loading profiles to be applied to the plantar tissues.

## Materials and methods

An Ultrasound probe, Linear Variable Displacement Transducer (LVDT) and load cell were mounted in series within a cylindrical column. The column was driven vertically by an actuator to contact the plantar surface of the heel. Drive profiles were generated from barefoot walking motion data and input to the actuator. Output from the load cell and LVDT were recorded at 3kHz. Ultrasound images were recorded at 200Hz. The subject’s leg was braced such that tissue strain of 0.4 (tissue thickness of 10.66mm) was predicted when vertical displacement of the device peaked. Three trials of 10 cycles were conducted for 1 subject to assess device motion compared to tissue compression.

**Figure 1:**
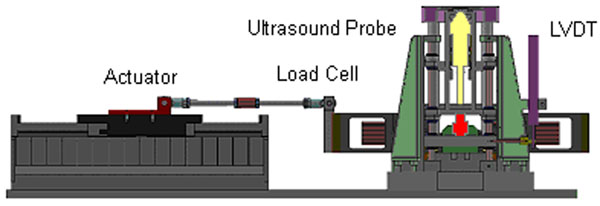
STRIDE

## Results

**Figure 2:**
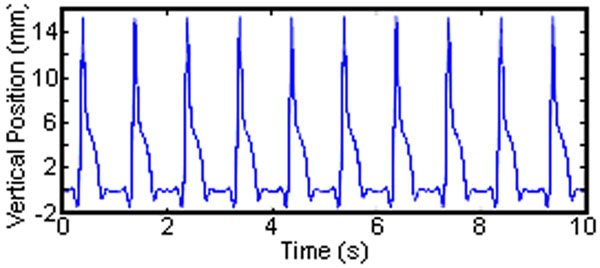
Measured vertical displacement

**Figure 3:**
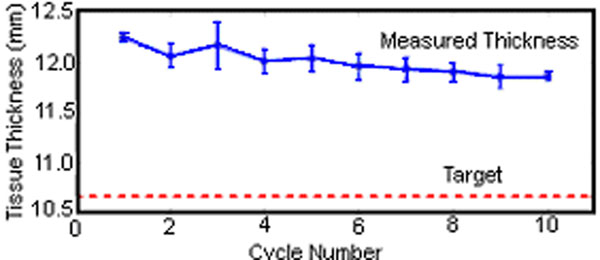
Tissue thickness at peak compression

Measured vertical displacement (Fig2) matches the input motion profiles (ICC>0.99) for all cycles. Minor deviation occurred at peak accelerations (loading/unloading transition) but reduced over multiple cycles with a final error of +0.19mm (SD, 0.01). The measured tissue thickness at peak compression was greater (mean, +1.34mm; SD, 0.13) than the target thickness (10.66mm), however showed a reduction from 12.24mm to 11.85mm after 10 cycles (Fig3).

## Conclusions

STRIDE replicated the rapid loading experienced by the heel during gait. The ultrasound, load cell and LVDT measured the tissue response to compression. To achieve target strains improved bracing and multiple compressions are required.
